# Low back pain during military service predicts low back pain later in life

**DOI:** 10.1371/journal.pone.0173568

**Published:** 2017-03-10

**Authors:** Ville M. Mattila, Heikki Kyröläinen, Matti Santtila, Harri Pihlajamäki

**Affiliations:** 1 Research Department, Centre for Military Medicine, Helsinki, Finland; 2 Department of Orthopedics and Traumatology, Tampere University Hospital, Tampere, Finland; 3 Coxa Joint Replacement Hospital, Tampere, Finland; 4 Department of Biology of Physical activity, University of Jyväskylä, Jyväskylä, Finland; 5 Department of Military Pedagogy and Leadership, National Defense University, Helsinki, Finland; 6 Department of Orthopedics and Traumatology, Seinäjoki Central Hospital, Seinäjoki, Finland; 7 Faculty of Medicine and Life Sciences, University of Tampere, Tampere, Finland; Saint Louis University, UNITED STATES

## Abstract

The aim of the present study was to assess associations between physician diagnosed unspecified low back pain (LBP) during compulsory military service and self-reported LBP and physical fitness measured on average four years after military service. From a total of 1155 persons who had been pass medical examination for military service and who had completed physically demanding military training between 1997 and 2007, 778 men participated in a refresher military training course and physical tests. In this study, the association between LBP during military service and LBP in later life in addition to the association between LBP and physical fitness were examined. A total of 219 out of 778 participants (28%) had visited a physician due to some musculoskeletal symptom (ICD-10 M-diagnosis) during their military service. Seventy-four participants (9.5%) had visited a physician due to unspecified LBP during their service, and 41 (5.3%) had temporarily been absent from duty due to LBP. At the follow-up examination, 122 (15.7%) had reported LBP during the past month. LBP during military service was associated with self-reported LBP in the follow-up (p = 0.004). Of those who had been absent from duty due to LBP during their military service, 13 (31.7%) reported LBP during the past month. In risk factor analysis, no initial health behaviour and physical performance variables were associated with baseline LBP in the follow-up. The main finding of the present study was that unspecified LBP during military service predicts LBP in later life. On the basis of previous literature, it is also known that LBP is a common symptom and thus, one cannot expect to be symptomless the entire life. Interestingly, none of the health behaviours nor the physical performance studied in the follow-up were associated with baseline LBP. It appears that individuals prone to LBP have symptoms during physically demanding military service and also later in their life.

## Introduction

Deconditioning caused by physical inactivity has aroused concern in Western societies. Physical inactivity has been associated with several chronic diseases of which non-specific low back pain (LBP) is one of the most predominant [1]. Low back pain is defined as pain in the lumbar or gluteal region with or without radicular pain to the lower extremities. The vast majority of individuals with pain affecting the lower back has no specific diagnosis and is categorized as having non-specific LBP [2], i.e. the cause of non-specific LBP is usually unknown. Non-specific LBP is common in adolescence [3–6], and the occurrence of LBP increases with age until adulthood. Moreover, there is strong evidence to suggest that adult LBP originates in adolescence [3]. Low back disorders are the most prevalent musculoskeletal health concerns in populations and can cause varying degrees of disability [7]. In adult populations, psychosocial difficulties [8, 9], smoking [10], overweight [10], sleep disturbances [11], and poor self-rated health [9] have been suggested to be risk factors for LBP leading to increased disability. LBP is also a very common disorder among military personnel causing disability, lost worker productivity, and increased health care costs [12].

Low back pain is a common condition worldwide, and it has been estimated that its prevalence will increase substantially in future [13, 14]. A systematic review of population-based studies estimated the global point prevalence of LBP to be 12%, with a 1-month prevalence of 23% [14]. The overall mean prevalence was 31%, the one-year prevalence was 38%, and the lifetime prevalence was 40%. Women had significantly higher point and annual prevalence; although no gender differences were seen for the 1-year or lifetime prevalence of LBP [14]. The prevalence of LBP did not increase in men after 40 years of age, while the prevalence peaked later among women. In addition, LBP has been reported to be as prevalent in middle-aged populations as among those aged 60 years or over, but the prevalence seems to decline among the oldest individuals [15]. Another systematic review concluded that the prevalence of severe forms of back pain continues to increase with age, whereas less severe back pain becomes less common after reaching a peak when the individual is 50 to 60 years of age [16]. Moreover, there are some reports that suggest the prevalence of non-specific LBP decreases with age [17, 18].

The relation between physical activity and LBP has been demonstrated to be controversial because both high [19–21] and low amounts and / or intensities [22, 23] of physical activity have been recognized as risk factors for LBP. In addition, it has been shown that individuals with chronic LBP have reduced physical fitness levels compared with healthy asymptomatic subjects [24]. Moreover, it has been stated that chronic LBP sufferers have lower aerobic capacity and a higher fat percentage than their healthy controls [25]. It should be pointed out, however, that these findings mainly originate from a cross-sectional study setting, and thus may be biased. The question whether deconditioning is the cause of LBP or whether LBP contributes to physical inactivity has been raised [26].

Low back pain leads to care-seeking behaviour and reduced health-related quality of life in adolescence [27]. Furthermore, LBP in late adolescence has been reported to predict LBP in adulthood [28, 29]. LBP is believed to be prevalent throughout life and is often recurring [30, 31]. When assessed for pain intensity, quality of life, disability, or health care utilization, patients with radiating LBP tend to have a poorer prognosis when compared with patients suffering from non-radiating LBP [32].

The relationship between physical activity levels and LBP was recently assessed in a review article by Hendrick and coworkers [33]. In the review article, twelve studies were identified, of which five were cross-sectional ones. They suggest that physical activity and LBP may not be associated. However, they state that there is a need for high-quality longitudinal studies. Thus, the aim of this present study was to examine the association between physician diagnosed un-specified LBP in healthy males during compulsory military service, self-reported LBP, and physical fitness levels measured on average four years after military service. We hypothesize that LBP during military service predicts lower levels of physical fitness and LBP in later life.

## Subjects and methods

In Finland, military service lasting from 6 to 12 months is compulsory for all male citizens above 18 years of age, and approximately 80% of Finnish males complete military service. The special characteristics of military training are the intensity and the volume of physical training and activities, since one of the main goals of the training is to improve the physical performance of conscripts. Because military service in Finland is compulsory, the epidemiological figures can be generalized quite well to the young adult male population.

As described in detail previously [34], military service begins with an 8-week basic training (BT) period comprising 135 hours of various types of physical training. In addition, conscripts also perform 56 hours of programmed physical training during the BT period. During combat training, every conscript must complete long marches carrying personal combat gear weighing 25–35 kg. After the BT period, the amount of moderate and high-intensity programmed physical training is slightly reduced, but the intensity and volume of the marches carrying heavy combat gear increase.

During military service, all conscripts can use the medical services provided by military health care and hospitals. After careful clinical examination and the necessary diagnostic tests and imaging, the most accurate diagnosis is selected by a physician according to the 10th Revision of the International Classification of Diseases and Related Health Problems (ICD-10). All visits by conscripts to a military physician due to unspecified LBP with the ICD-10 diagnostic code M54.5, M54.4 or M54.9 are recorded. For the present study, a computer search using these ICD-10 diagnostic codes was conducted. The original, completed medical records were retrieved and reviewed to confirm the accuracy of the diagnoses and to systematically collect data for the present study. LBP that occurred during the conscript's leisure time or on the way from or to the garrison was included in the collected data. The length of absence from duty due to LBP was also recorded.

The subjects selected for this study were 1155 persons who had passed the entry medical examination for military service and who had completed physically demanding military training between 1997 and 2007. Of these, 920 participated in a refresher military training course. The most common reasons for nonparticipation in the refresher course were work, study, or health-related issues. From a total of 920 males, 778 volunteered (mean ± SD age 19.9±4.6 yrs., height 1.80±0.06m, body mass 80.3±13.4kg, and body mass index 24.7±3.8) to take part in the present study. The mean follow-up period, the period between the end of military service and participation in the refresher course, of the subjects was approximately four years (ranging from one to eleven years). The subjects were informed of the study protocol and written informed consent was obtained. The ethical statement of the present study was given and approved by the ethical committee of the Central Finland Health Care District, Jyväskylä, Finland (K-S shp:n Dnro 34/2007, 21.8.2007).

### Questionnaire

Measurements were carried out in 8 different sessions during the refresher course in 2008 (March-November). During the refresher course, subjects completed a questionnaire that included questions on socioeconomic background, physical activity, and health behaviours. The urbanisation level of residence was determined by population density using four categories: city/large town (population over 90,000), small town, village (densely populated area in rural municipalities), and sparsely populated rural municipality (isolated homestead in rural municipalities). Three categories of achieved level of education were used: comprehensive school, vocational school and upper secondary school, or university. Marital status was categorised as single or other. Self-estimated health status was categorised as good, average, and poor. Smoking and alcohol consumption habits were assessed by questions about daily smoking and the frequency of consumption of more than 6 units of alcohol per day (less or more frequently than once a month). LBP was assessed with two questions: 1) Assess how many days all together you have had LBP that has radiated to the lower extremity below the knee during the last month? Alternatives: None, 1–7 days, 8–14 days, more than 14 days but not daily, daily. 2) Assess how many days all together you have had acute LBP (e.g. lumbago) during the last month? Alternatives: None, 1–7 days, 8–14 days, more than 14 days but not daily, daily. LBP during the last month (yes/no) and the intensity of LBP during the last week were studied using the Visual Analogue Scale (VAS).

### Physical fitness tests

Maximal oxygen uptake (VO_2_max) was indirectly predicted during a bicycle ergometer test (Ergoline 800 S, Ergoselect 100 K or 200 K, Bitz, Germany) [35]. After a 5-min warm up, the test began with a power output of 75 W, which was increased by 25 W after every second minute. The pedalling rate of 60 rpm was maintained constant throughout the test. The heart rate (HR) was recorded continuously (Polar Vantage NV or S610, S710 or S810, Kempele, Finland). The test was terminated at volitional exhaustion. Predicted VO_2_max was determined from HR and power (Fitware, Mikkeli, Finland) as follows: VO_2_max (ml·kg^-1^·min^-1^) = 12.35*P_max_/kg + 3.5, where P_max_ is maximal power in relation to body mass. During their military service, they had also run the 12-min running test. Conscripts were instructed to perform the test with a maximal effort but at progressively increasing running speed. The accuracy of the measurements was ±10 meters.

Muscle fitness was measured by tests of grip strength, sit-ups, push-ups, repeated squats, and maximal isometric leg and arm extensions. Isometric grip strength was measured twice in a sitting position (90° elbow angle) with a dynamometer (Saehan Corporation, Masan, South Korea). The best results for the right and left hands were averaged for the final outcome [36]. The results of the push-ups, sit-ups, and repeated squats were expressed as the number of correctly performed repetitions within 60 s. The detailed descriptions of these tests have been published earlier [37].

Maximal isometric leg and arm extension forces (leg and bench press) were measured bilaterally using dynamometers. Knee angle was set to 107°. During the maximal bench press, participants were in a supine position on a bench with their feet on the floor and their elbows positioned at an angle of 90°. A total of three maximal trials were performed with a 30 s recovery period between trials. The best performance recorded was included for further analysis. The participants were also instructed to produce maximal strength as fast as possible and to maintain it for 3 s. Maximal force and force production time were collected with an AD-converter (CED power 1401, Cambridge Electronic Design, Ltd, England) at a frequency of 1 kHz, on a computer [38]. More details on the testing procedures can be found in an article by Vaara et al. [38]

### Statistical analysis

During the first stage, two-way tables were calculated for our main variable (yes/no physician visit due to low back pain during military service) and the categorized variables measured during the refresher training course after a mean four-year follow-up. A chi-square test was used with the level of significance defined as p = 0.05. Next, separate adjusted (adjusted by age, urbanisation level of residence, family composition, level of education, smoking, drinking) logistic regression models for physical fitness and the LBP variables measured at the follow-up were calculated in order to assess whether physician diagnosed LBP during military service is associated with levels of physical fitness or LBP later in life. Odds ratios (OR) were estimated with 95% confidence intervals (95% CI).

Logistic regression analyses were only performed for respondents who provided answers to every question, and thus respondents with incomplete answers were excluded from the analysis. The frequency of missing values for the independent variables varied from 2 to 8%.

## Results

Of the 778 participants who had completed their military service between 1997 and 2006, 219 (28%) had visited a physician due to musculoskeletal symptoms (ICD-10 M-diagnosis) during their military service. The mean duration of absence from duty was 1.2 (range 1 to 44) days. Seventy-four (9.5%) conscripts had visited a physician due to unspecified LBP during their military service, and 41 (5.3%) had temporarily been absent from duty due to LBP. The mean duration of absence from duty due to LBP per physician visit was 2.6 days (range 1 to 6).

From the self-reported variables at the follow-up in 2008, level of education (p = 0.23), marital status (p = 0.31), alcohol consumption (p = 0.62), daily smoking (p = 0.54), or self-estimated perceived health (p = 0.33) were not associated with absence from duty due to LBP. In addition, the result of the 12-min running test during the military service was not associated to absence from duty due to LBP (p = 0.71).

When muscle strength and aerobic capacity in the follow-up and absence from duty due to LBP during military service were assessed (Table 1), it was observed that absence from duty due to LBP during military service did not predict later physical performance, including aerobic capacity and muscle fitness.

**Table 1:** Odds ratios for absence from military service due to low back pain.

Characteristics	Adjusted* OR
*Maximal oxygen uptake (l/min; by quartiles)*	
First (1.84 to 2.86)	1
Second (2.87 to 3.25)	1.2 (0.4–3.6)
Third (3.26 to 3.68)	2.5 (0.9–7.2)
Fourth (3.69 to 5.73)	1.4 (0.4–4.8)
*Grip strength (kg; by quartiles)*	
First (31.5 to 46.5)	1
Second (46.6 to 52.0)	0.6 (0.3–1.7)
Third (52.1 to 58.5)	1.0 (0.4–2.3)
Fourth (58.6 to 85.5)	0.8 (0.3–2.0)
*Sit-ups (reps/min; by quartiles)*	
First (2 to 31)	1
Second (32 to 39)	1.0 (0.4–2.2)
Third (40 to 45)	1.1 (0.4–2.9)
Fourth (46 to 72)	0.7 (0.2–2.2)
*Push-ups (reps/min; by quartiles)*	
First (1 to 19)	1
Second (20 to 27)	0.9 (0.4–2.4)
Third (28 to 38)	0.9 (0.3–2.3)
Fourth (39 to 75)	0.4 (0.1–1.4)
*Repeated squats (reps/min; by quartiles)*	
First (3 to 39)	1
Second (40 to 44)	0.6 (0.2–1.7)
Third (45 to 50)	0.9 (0.4–2.2)
Fourth (51 to 64)	0.8 (0.3–2.1)
*Maximal leg extension (kg; by quartiles)*	
First (89 to 235)	1
Second (236 to 277)	0.8 (0.3–2.1)
Third (278 to 337)	1.1 (0.4–2.8)
Fourth (338 to 740)	0.9 (0.3–2.4)
*Maximal bench press (kg; by quartiles)*	
First (48 to 76)	1
Second (77 to 87)	0.8 (0.3–2.0)
Third (88 to 110)	0.6 (0.2–1.6)
Fourth (111 to 163)	1.1 (0.5–2.7)

*Adjusted for age, BMI, level of education, marital status, drinking and daily smoking

Of the 788 respondents in the follow-up examination, 122 (15.7%) had reported LBP during the past month. LBP during military service was associated with self-reported LBP in the follow-up (p = 0.004). Of those who had been absent from duty due to LBP during military service, 13 (31.7%) reported LBP during the past month while the corresponding figure was 109 (14.8%) among those who had no LBP absence from duty.

In the follow-up, LBP during the month before the refresher military training course was not associated with temporary absence from duty due to LBP during one-week physically strenuous military training (p = 0.71). The mean LBP VAS during the military training week was 1.5 for those who had no absence from duty due to LBP and 1.9 for those who had absence from duty due to LBP. However, these results were not statistically significant (p = 0.17).

## Discussion

The main finding of the present study was that unspecified LBP during military service predicts LBP in later life. An earlier study has shown that conscripts who suffer from chronic LBP before entering military service have a ten-fold higher risk of experiencing LBP during military service compared to the risk before military service [39]. A history of suffering from LBP seems to predict a later LBP episode. Thus, young men who suffer LBP during their military service should not be directed towards occupations that require a symptomless low back without special rehabilitation and muscle fitness training. According to the findings of Suni et al., the risks for LBP can be reduced by education and muscle fitness training [40]. They noticed that exercise and education improved the control of the lumbar neutral zone. This could have a prophylactic effect on LBP-related off-duty service days in the military environment when implemented as part of military service among young healthy men. However, more research data and knowledge are also needed to establish how physical activity and fitness associate with LBP.

The one-month occurrence of LBP in our study (16%) was lower than one-month occurrence described previously in a review article (23%) [14]. This is probably due to our healthy subjects that had passed physically demanding military service. In addition, they described the mean VAS scale to be 1.5 to 1.9 which can be considered to be relative low. Interestingly, none of our background variables, health behaviour, or the physical performance at the follow-up was associated with baseline LBP in the present study. Thus, focusing LBP preventive measures on those variables does not seem to be useful. A previous randomized controlled study on the effect of using orthotic insoles during military service concluded that orthotic insoles should not to be used with the aim of toward preventing LBP episodes in young male adults [41]. A previous longitudinal population-based study found that being overweight or obese in early adulthood as well as during later years increases the risk of radiating but not non-specific LBP among men [42]. The results of the present study also contradict the findings of Taanila et al. [43] who found that the risk for LBP increased among young men who had a lower educational background and lower levels of both aerobic and muscular performance.

The compulsory military service for all Finnish male citizens above 18 years of age differs from professional armies. In a conscription army, the volume and intensity of physical training have to be carefully adjusted to the conscripts’ fitness level. Therefore, the present results cannot be directly extrapolated to professional armies. However, due to the obligatory nature of military service in Finland, the epidemiological figures can be generalized quite well to the young adult male population because approximately 80% of men complete the 6 to 12 month military service period.

The strengths of the present study include the fact that the original cohort of conscripts comprised a significant number of individuals who were obliged to use the medical services provided by the Finnish Defence Forces for the management of LBP. Furthermore, all conscripts had passed two physician-performed medical examinations when entering military service. Thus, all patients with severe back diseases, such as significant scoliosis or other congenital back anomalies, severe post-traumatic disorders, rheumatoid arthritis, or ankylosing spondylitis were exempted from duty. Conscripts can thus be considered healthy young men. The accuracy and validity of the LBP during conscription is excellent, as the cohort included in this study had no alternative choices such as private clinics to attend due to LBP. Moreover, the authors conclude that the present results can very well be generalized to a young healthy male population owing to the compulsory nature of Finnish military service.

The present study also has some weaknesses. A limitation of the study is that although subjects were healthy men, their previous LBP episodes, level and recurrence before entering the military service was not known. Unfortunately, during the service it was not possible to identify less severe LBP episodes that did not result in a visit to a physician during conscription. Furthermore, only 778/1155 participated in the refresher course which provides a limitation to the study, since the prevalence of LBP in those not attending the course is not known. In addition, a mean follow-up period of four years was the midterm follow-up duration, but this time-frame was chosen due to the requirements of military service.

The main finding of the present study was that unspecified LBP during military service predicts LBP in later life. On the basis of previous literature, it is also known that LBP is a common symptom and thus, one cannot expect to be symptomless the entire life. Interestingly, none of the health behaviours nor the physical performance studied in the follow-up were associated with baseline LBP. It appears that individuals prone to LBP have symptoms during physically demanding military service and also later in their life.
